# Data transformation

**DOI:** 10.4103/0976-500X.72373

**Published:** 2010

**Authors:** S Manikandan

**Affiliations:** *Assistant Editor, JPP*

Preparing the data facilitates statistical analysis and this includes data checking, computing-derived data from the original values, statistically adjusting for outliers and data transformation. The initial three methods have been explained previously in this series.[1] Data transformation also forms part of initial preparation of data before statistical analysis.

## WHEN TO DO TRANSFORMATION?

The pattern of values obtained when a variable is measured in large number of individuals is called a distribution.[2] Distribution can be broadly classified as normal and non-normal. The normal distribution is also called ‘Gaussian distribution’ as it was first described by K.F. Gauss. This is called normal distribution as most of the biological parameters (such as weight, height and blood sugar) follow it. There are a very few biological parameters which do not follow normal distribution, for example antibody titre, number of episodes of diarrhoea, etc. The beginners should not be confused with the term ‘normal’ as it does not necessarily imply clinical normality and there is nothing abnormal in the ‘non-normal’ distributions.

One of the assumptions of the statistical test used for testing hypothesis is that the data are samples from normal distribution.[3] Hence it becomes essential to identify skewed/normal distributions. There are some simple ways to detect skewness.[4]

- If the mean is less than twice the standard deviation, then the distribution is likely to be skewed.
- If the population follows normal distribution, then the mean and the standard deviation of the samples are independent. This fact can be used for detecting skewness. If the standard deviation increases as the mean increases across groups from a population, then it is a skewed distribution.

Apart from these simple methods, normality can be verified by statistical tests like Kolmogorov - Smirnov test.

Once skewness is identified, every attempt should be made to convert it into a normal distribution, so that the robust parametric tests can be applied for analysis. This can be accomplished by transformation.

Transformations can also be done for the ease of comparison and interpretation. The classical example of a variable which is always reported after logarithmic transformation is the hydrogen ion concentration (pH). Another example where transformation helps in the comparison of data is the logarithmic transformation of dose-response curve. When the dose-response relationship is plotted it is curvilinear. When the same response is plotted against log dose (log dose-response plot) it gives an elongated S-shaped curve. The middle portion of this curve is a straight line and comparing two straight lines (by measuring their slope) is easier than comparing two curves. Hence transformation can assist in the comparison of data.

In a nutshell, transformation can be carried out to make the data follow normal distribution or at times for ease of interpretation/comparison.

## WHICH TYPE OF TRANSFORMATION TO USE?

Many a times, the transformation which makes the distribution normal also makes the variance equal. Even though there are many transformations like logarithm, square root, reciprocal, cube root, square, the initial three are more commonly used. The following are the guidelines for the selection of a method of transformation.[5]

- If the standard deviation is proportional to the mean, the distribution is positively skewed and logarithmic transformation is the ideal one.
- If the variance is proportional to the mean, square root transformation is preferred. This happens more in case of variables which are measured as counts e.g., number of malignant cells in a microscopic field, number of deaths from swine flu, etc.
- If the standard deviation is proportional to the mean squared, a reciprocal transformation can be performed. Reciprocal transformation is carried out for highly variable quantities such as serum creatinine.

Among these three transformations, logarithmic transformation is commonly used as it is meaningful on back transformation (antilog).[3,6]

### Caution

A small cautionary note for the beginners performing transformation is that all calculations should be done in the transformed scale and back transformation should be done only at the end.

Many researchers think that transformation of data is ‘data deceiving’. They are assured that transformation is a statistically approved method and it is universally valid.

## HOW TO REPORT?

While reporting the results, the summary statistics of the raw data should be mentioned. The transformation done should be clearly mentioned along with the reason for transformation. One should not forget to mention that all the statistical analyses were carried out on the transformed data.[7] Finally the back transformation value (especially for 95% confidence interval) should also be mentioned.
